# The Nasal Route as a Potential Pathway for Delivery of Erythropoietin in the Treatment of Acute Ischemic Stroke in Humans

**DOI:** 10.1100/tsw.2009.103

**Published:** 2009-09-15

**Authors:** Julio Cesar García-Rodríguez, Iliana Sosa Teste

**Affiliations:** National Center for Laboratory Animal Breeding (CENPALAB), Havana, Cuba

**Keywords:** erythropoietin, stroke, nasal delivery, neuroprotection

## Abstract

Intranasal delivery provides a practical, noninvasive method of bypassing the blood-brain barrier (BBB) in order to deliver therapeutic agents to the brain. This method allows drugs that do not cross the BBB to be delivered to the central nervous system in a few minutes. With this technology, it will be possible to eliminate systemic administration and its potential side effects. Using the intranasal delivery system, researchers have demonstrated neuroprotective effects in different animal models of stroke using erythropoietin (EPO) as a neuroprotector or other different types of EPO without erythropoiesis-stimulating activity. These new molecules retain their ability to protect neural tissue against injury and they include Asialoerythropoietin (asialoEPO) carbamylated EPO (CEPO), and rHu-EPO with low sialic acid content (Neuro-EPO). Contrary to the other EPO variants, Neuro-EPO is not chemically modified, making it biologically similar to endogenous EPO, with the advantage of less adverse reactions when this molecule is applied chronically. This constitutes a potential benefit of Neuro-EPO over other variants of EPO for the chronic treatment of neurodegenerative illnesses. Nasal administration of EPO is a potential, novel, neurotherapeutic approach. However, it will be necessary to initiate clinical trials in stroke patients using intranasal delivery in order to obtain the clinical evidence of its neuroprotectant capacity in the treatment of patients with acute stroke and other neurodegenerative disorders. This new therapeutic approach could revolutionize the treatment of neurodegenerative disorders in the 21 century.

## INTRODUCTION

Stroke represents the third most common cause of death in industrialized countries and the foremost reason for disability in adults[1,2]. Therefore, therapy for acute ischemic stroke is of primary importance to clinical neuroscience in the 21$^{st}$ century. For more than 20 years, neuroscientists have searched for a drug that protects ischemic brain tissue from cell death. The reality is that over a thousand compounds have been tested in animal models of ischemic stroke, but of the compounds that have successively entered clinical trials, none has been successful in clinical practice, until now[1,3].

The hope of having a drug of universal use that cures stroke is, without a doubt, an unreachable utopia for some investigators, thus questioning the role of neuroprotection in brain ischemia[4,5,6]. Systemic erythropoietin (EPO) is a hematopoietic growth factor that regulates red blood cell production. Its action consists of inhibiting apoptosis of erythrocytic progenitors[6]. Independently of its hematopoietic actions, EPO and EPO variants are directly neuroprotective in *in vitro* and *in vivo* ischemic models[7]. However, a potential risk is always present when using EPO systemically. EPO might worsen the outcome after stroke either because of hematocrit elevation or other negative effects[8].

The purpose of this review is to outline the possibility of using EPO or modified EPO, which includes Asialoerythropoietin (asialoEPO)[9,10], carbamylated EPO (CEPO)[11,12,13], and rHu-EPO with low sialic acid content (Neuro-EPO)[14,15] as a neuroprotector, as well as the advantages of the nasal administration of this cytokine. The low sialic acid content of Neuro-EPO makes it very similar to the one that occurs in the mammalian brain[16], which has been documented as an endogenous neuroprotector for cerebral ischemia and other brain injuries.

## rHu-EPO AS A NEUROPROTECTANT

A drug that is sufficiently effective, with safe access to the central nervous system (CNS), has not yet been developed for neuroprotective treatment in acute or chronic stages of neurological diseases.

As most of the neuroprotective agents effective in ischemia biomodels have failed to be clinically tolerated[17,18,19], a strategy to circumvent this problem could be the use of the same molecules expressed in the brain after different lesions[20,21], thus helping in the maintenance of homeostasis. One of these molecules is EPO.

EPO is a kidney-produced glycoprotein involved in proliferation, differentiation, and maturation of erythrocytes and other hematopoietic cells, increasing oxygen supply to tissues[22,23,24].

The study of the neuroprotective effect of EPO has been stimulated by the fact that this chemical is normally expressed within the brain and is regulated by hypoxia inducible factor 1 (HIF-1)[9,25], which is, in turn, activated by a wide variety of stress factors. Different *in vitro* models and several models of stroke have been used for this purpose[26,27,28,29].

The neuroprotective efficacy of rHu-EPO has been tested in several animal models of nervous system injury (mouse, rat, gerbil, and rabbit), including focal and global cerebral ischemia, showing a reduction of neuronal death, a better outcome, and a significant decrease of brain infarct volumes[13,30,31,32].

Although the neuroprotective mechanism of rHu-EPO is still being investigated, it is known that this effect is mediated by receptors located at the walls of the vascular endothelia and astrocytes[13,31].

The neuroprotective mechanism of rHu-EPO seems to be multifactorial. The rHu-EPO molecule positively modulates the expression of antioxidant enzymes and reduces nitric oxide–mediated formation of free radicals, by a mechanism involving JAK2 and the nuclear factor NF-κB. Its antioxidant action is also sustained by restoring cytosolic catalase and glutathione peroxidase activities in erythrocytes, which protects against oxidative stress by reducing lipid peroxidation[33,34].

It has been demonstrated that rHu-EPO also displays neurotrophic activity, which implies an effect of larger latency than the inhibition of apoptosis[34,35] and reduces neuronal excitotoxicity involved in many forms of cerebral injury. rHu-EPO has also been identified as a potent mediator of tolerance to ischemia[36,37].

As with other HIF-1–induced cytokines, this glycoprotein promotes angiogenesis as a response to hypoxia and neuronal injury[38,39,40] by stimulating the generation of microvessels through the interaction with its receptor in the blood vessels[37].

Its antiapoptotic action is given through the rHu-EPO–mediated activation of JAK2, which, in turn, leads to the activation of NF-κB and to the overexpression of the apoptosis-inhibiting genes XIAP and c-IAP2[35,38,40,41]. rHu-EPO protects neurons from ischemic injury by overexpression of Bcl-x in the hippocampus of gerbils[33]. rHu-EPO inhibits the expression of Bax in PC12 cells and increases the expression of Bcl-XL, a member of the group of Bcl-2 antiapoptotic proteins[33,40,42].

At the same time, it stimulates cell survival by inhibiting the MAPK and PI3K/Akt complex that promotes apoptosis[33]. These data suggest that rHu-EPO acts by controlling the balance of the expression of either pro- or antiapoptotic molecules[24].

The neuroprotective effect attributed to rHu-EPO can also be derived from its anti-inflammatory effect[41]. The administration of rHu-EPO to rats with focal ischemia reduces the migration of inflammatory cells to the ischemic tissue notably, attenuating the production of proinflammatory cytokines and limiting the size of the lesion[4,31,33]. The expression of EPO is markedly reduced by proinflammatory cytokines and by oxygen reactive species[43,44,45]. This could contribute to the action of these mediators in the pathogenesis of ischemia and explain why exogenous administration of rHu-EPO can be especially beneficial[46].

It has also been proposed that rHu-EPO could exert its anti-inflammatory effect by inhibiting molecular signaling in the injured neurons[68]. The anti-inflammatory cell protection activity of rHu-EPO has been demonstrated in *in vitro* experiments with glial and neuronal cell cocultures, where neuronal death is associated with the release of TNF by glial cells[38,47,48].

### Stroke Clinical Trial with rHu-EPO and Derivatives

Considering the properties of rHu-EPO, Ehrenreich and coworkers carried out the first clinical trial with intravenous administration of this drug in acute cerebral ischemia, with a therapeutic window within the first 8 h. The authors reported a significant reduction of the infarct area in treated patients, associated with a noteworthy neurological and clinical improvement 1 month after ischemia[49]. The increase of rHu-EPO levels in serum and cerebrospinal fluid (CSF) suggests that rHu-EPO can cross the damaged blood brain barrier (BBB) and protect from cerebral ischemic injury, as postulated by other investigators[50].

No severe side effects have been reported after rHu-EPO treatment in ischemia or other neurodegenerative diseases[51].

Recently, a Safety Study of CEPO to treat Patients With Acute Ischemic Stroke was conducted with good result[52]. No clinical trials have been reported using asialoEPO.

### Other Illnesses where rHu-EPO has been Applied

The use of rHu-EPO in animal models of subarachnoid hemorrhage[53], intracranial hemorrhage[54], brain trauma[55,56], and spinal cord injuries[57,58] has been reported. It has also been reported that this drug reduces neuronal functional damage in models of encephalitis and multiple sclerosis[59,60], and improves diabetic neuropathy[61] and retinal ischemia[62]. Moreover, rHu-EPO has been used to treat cases of schizophrenia[63] and perinatal hypoxia[64,65,66]. Due to its stimulatory effects on neuronal plasticity, the use of rHu-EPO could have effects on the long-term recovery of patients with disabilities in cerebral vascular diseases or neurodegenerative disorders[67].

## EPO TARGET IN BRAIN TISSUE

The EPO receptor (EPO-R) belongs to the class I cytokine receptor superfamily, which is a group of class I transmembrane proteins that share certain structural features; in particular, four conserved cysteine residues and the WS x WS motif in their extracellular domain.

It has been demonstrated that EPO and EPO-R are expressed in brain tissue and their expression increases during ischemia, suggesting that they are involved in an endogenous neuroprotective system in the mammalian brain[50,68,69]. Recent observations suggest that an additional receptor for EPO mediates tissue protection. This receptor is pharmacologically different from that of erythropoiesis because it has a lower affinity for EPO and forms distinct molecular species in cross-linking experiments[70]. The receptor that promotes tissue protection is a heteromer composed of EPO-R and CD131, the β common receptor (βcR)[70]. CD131 also forms receptor complexes with the α receptor subunits specific for granulocyte-macrophage colony stimulating factor (GM-CSF), IL-3, and IL-5, and it has been termed the "*common*" receptor (see review by Murphy and Young[71]).

## NEW NONERYTHROPOIETIC MOLECULES WITH NEUROPROTECTANT ACTIVITY

Recently, several different types of new EPOs without erythropoiesis-stimulating activity have been developed. These new molecules retain their ability to protect neural tissue against injury; they include Asialoerythropoietin (asialoEPO)[9,10], carbamylated EPO (CEPO)[11,12,13], and rHu-EPO with low sialic acid content (Neuro-EPO)[14,15].

## AsialoEPO

The need for nonerythropoietic rHu-EPO derivatives that still retain neuroprotective action has led to the discovery of asialoEPO, generated by total enzymatic desialylation of rHu-EPO. AsialoEPO has the same EPO-R affinity and neuroprotective properties as EPO, but an extremely short plasma half-life[72].

The ability to dissociate the tissue-protective actions of EPO from its erythropoietic actions may eventually be applied in the clinic to promote neurological regeneration without increasing red blood cell formation[72]. Erbayraktar and coworkers[72] have shown protective activities of the nonerythropoietic asialoEPO in models of cerebral ischemia, spinal cord compression, and sciatic nerve crush. Additionally, asialoEPO protects against neonatal hypoxia-ischemia as potently as EPO in hypoxic-ischemic brain injury in 7-day-old rats[10].

## CEPO

Another modified EPO molecule that solely manifests tissue-protective action without erythropoietic activity may have a more targeted effect. As an example, transformation of lysine to homocitrulline by carbamylation gives rise to carbamylated EPO (CEPO)[52].

CEPO, similar to asialoEPO, lacks erythropoietic effect, but still shows neuroprotective effects in animal models of stroke, diabetic neuropathy, and experimental autoimmune encephalomyelitis to an extent comparable to that of EPO[73]. It is important to note that CEPO has a minimal affinity for EPO-R and that its effects are mediated via a different EPO receptor. It is thought that this receptor consists of the EPO-R monomer together with a dimer of the common receptor (βCR)[74]. Recently, an investigation carried out in a rat model of focal cerebral ischemia showed that postischemic intravenous treatment with CEPO led to improved functional recovery[75]. In 2007, a study by Mahmood et al. assessed the effect of intraperitoneally infused rHu-EPO and CEPO in a traumatic brain injury rat model, and they concluded that rHu-EPO and CEPO are equally effective in enhancing spatial learning and promoting neural plasticity, but hematocrit was significantly increased only with rHu-EPO[76]. Similarly, a recent study by Wang et al.[7] demonstrated equivalent effects of rHu-EPO and CEPO in the reduction of neurological impairment in rats subjected to embolic middle cerebral artery occlusion (MCAO). As expected, rHu-EPO, but not CEPO, produced a transient increase in hematocrit levels. A neuroprotectant effect on spinal cord hemisection in the rat was also demonstrated[77].

Recently, another advantage of EPO over CEPO was demonstrated in rodents. Short-term treatment with EPO at doses optimal for neuroprotection caused significant alterations in platelet function and composition with *in vivo* hemostatic consequences, while CEPO treatment had no effect on these parameters[11].

## Neuro-EPO

During the biotechnological production of rHu-EPO, various isoforms with different contents of sialic acid are obtained. When the sialic acid content is 4–7 mol/mol protein, it is considered a low sialic acid–containing EPO (Fig. 1a). EPO, modified to display low sialic acid content (Neuro-EPO) is very similar to the one that occurs in the mammalian brain[16]. Low sialic acid–containing EPOs are rapidly degraded by the liver. Thus, this molecule could be administered by a nonsystemic route, such as the intranasal route, to prevent its hepatic degradation. The intranasal administration of Neuro-EPO has been shown to be safe; the molecule reaches the brain rapidly, does not stimulate erythropoiesis after acute treatments, and shows efficacy in some rodent models of brain ischemia and in nonhuman primates. This proposal could be considered a therapeutic option for stroke[14,15,78].

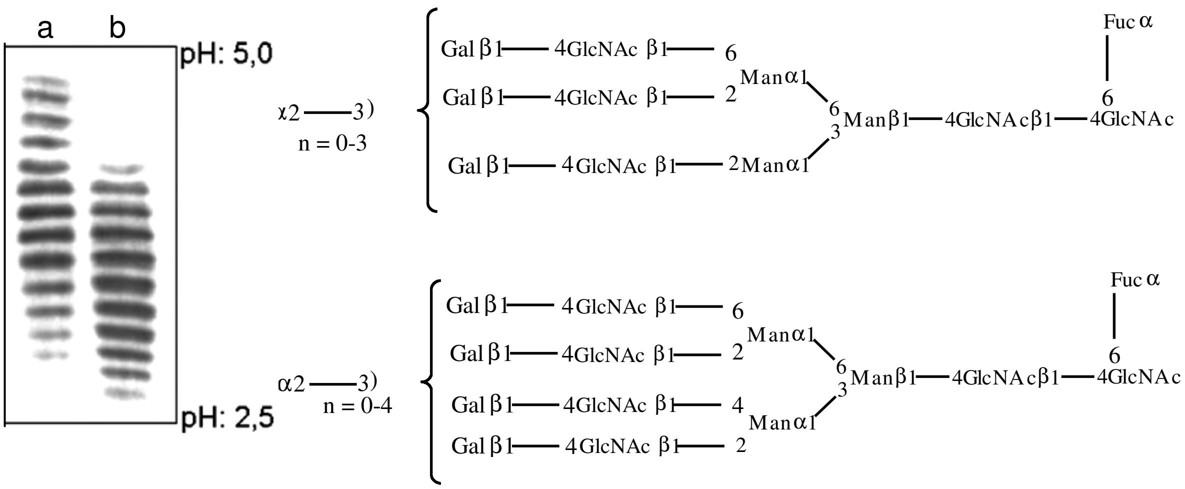

**FIGURE 1.** Differences between rHu-EPO and Neuro-EPO by isoelectric focusing method. Most abundant N-glycans covalenty linked to EPO molecule (AS): sialic acid. (a) Neuro-EPO: sialic acid content is 4–7 mol/mol protein. (b) rHu-EPO: sialic acid content is 10–12 mol/mol protein.

Contrary to the other EPO variants (asialoEPO and CEPO), Neuro-EPO does not present any chemical modification that differentiates it biologically from endogenous EPO. This constitutes a potential advantage over the other variants of EPO for the chronic treatment of neurodegenerative illnesses[52,79,80].

In summary, we can point out that EPO and EPO-derived compounds may have the potential to benefit different injuries of the CNS. For example, the use of a stroke therapy that alters elements of hemostatic function may interfere with the function of other anti- or prothrombotic drugs administered to these patients, thereby complicating their clinical management. Thus, CEPO, asialoEPO, and Neuro-EPO, with neuroprotective potential and devoid of hemostatic effects, may be beneficial as novel therapeutics in the treatment of stroke in the years to come.

## NASAL DELIVERY OF THERAPEUTIC MACROMOLECULES TO THE BRAIN

Delivery of therapeutic drugs from the nasal cavity to the brain (see review from Illum[81]) clearly involves extracellular pathways[82] as it occurs within minutes rather than hours[14]. Many therapeutic proteins have been successfully delivered to the brain using intranasal administration in different animal models: growth factors, such as NGF[46], interferon[83], insulin-like growth factor-I[84], and EPO in small rodents[14,17,85] and in nonhuman primates (*Maccaca fascicularis*)[86].

Intranasal drug administration could offer a noninvasive method for the treatment of acute or chronic brain insults, as it circumvents the BBB, providing rapid drug absorption, and eliminating its liver peripheral removal, without systemic side effects[81,87].

The BBB is a system of layers of cells at the cerebral capillary endothelium, the choroid plexus epithelium, and the arachnoid membrane, which are connected by tight junctions and which together separate the brain and the CSF from the blood[86,88,89].

We can say that the BBB has many properties similar to a continuous cell membrane, allowing lipid soluble molecules to traverse the membrane, while hydrophilic solutes demonstrate minimal permeation[88,90].

Indeed, the BBB limits the use of many therapeutic proteins developed by the biotechnological industry (e.g., human recombinant neurotrophic factors, cytokines, and other agents) with therapeutic potentiality to protect brain cells from acute or chronic damage and promote nerve repair.

Increasing interest has been placed in the possibility of circumventing the BBB by delivering proteins with therapeutic activity to the CNS through the intranasal pathway. This pathway is believed to involve two general mechanisms[91]. The first is internalization of the drug into the primary neurons of the olfactory epithelium, either by endo- or pinocytotic mechanisms, and intracellular axonal transport to the olfactory bulb. The second is an extracellular pathway that allows rapid absorption of the drug across the olfactory epithelial cells, either by trans- or paracellular mechanisms, followed by uptake into the CNS[92]. The trigeminal pathway following intranasal administration should not be excluded[93].

## Intranasal Delivery of rHu-EPO

Recently, a very small number of scientific papers have been published about different models of stroke that have used the nasal route to deliver EPO or EPO derivatives to the brain quickly and safely. A summary of these is listed in Table 1. The results reported by different authors agree and complement themselves; delivering EPO to the brain via the nasal route by avoiding the BBB. This pathway could be safer and 10 times faster than the intravenous route. The effects of EPO in the olfactory receptors and other potential unwanted side effects are unknown to the present.

The results reported with Neuro-EPO as a neuroprotectant have been very similar to those reported for the original rHu-EPO or its asialylated and carbamylated derivatives in ischemia models in Mongolian gerbils and rats[72,94]. The attenuation of learning disabilities induced by ischemia could be explained by EPO's effect of improving synaptic transmission during ischemia. This has been demonstrated in *in vitro* models[95]. It has also been postulated that rHu-EPO stimulates neuronal functionality by the activation of Ca++ and the release of neurotransmitters[27].

Some researchers have proposed that EPO can facilitate certain mechanisms of restoration and neuroplasticity in ischemic animals[67]. If true, this would open new therapeutic possibilities to stimulate tissue regeneration and recovery of brain areas by using a safe and noninvasive method, such as the intranasal route. As it has been described before, the olfactory region has unique physiological and anatomic attributes that define extracellular and intracellular routes to CNS, evading the BBB[96,97]. Nervous terminals responsible for conducting odor-related information lie in the upper part of the nasal cavity. The bundle of nerve cords that constitute the olfactory tract of the CNS passes through the holes of the cribiform plate and extends from the base of the brain to different subcortical regions. Consequently, small amounts of Neuro-EPO administered by this route quickly go into the brain and diffuse through the interstitial fluid. Therefore, the intranasal route can be used for prophylaxis and therapy of the CNS[82].

Several substances penetrate into the CNS by inhalation, and the passage of trophic and neuroprotective molecules through this route has been demonstrated[84].

When Neuro-EPO is administered by the intranasal route, it does not pass through the liver, where it would be rapidly degraded by proteases before reaching the CNS because of its low sialic acid content.

**TABLE 1**
**Published Papers of Preclinical Work in Animal Models of Stroke using Intranasal rHu-EPO or Derivatives of rHu-EPO Lacking Erythropoietic Activity**

| Species | Models | Dose, Vol., and Frequency of Nasal rHu-EPO | Assays | Ref. |
|---|---|---|---|---|
| Mongolian gerbil | Global ischemia | **Neuro-EPO** 249.4 UI/10 µl/8 h/4 days | Efficacy | [103] |
| Rat | Focal ischemia (MCAO) | **rHu-EPO** 12 UI/20 µl one dose | Efficacy | [98] |
| Mongolian gerbil | Focal ischemia | **Neuro-EPO** 249.4 UI/10 µl/8 h/4 days | Pharmacokinetic/efficacy/ security | [14,15,85] |
| Nonhuman primate (*M. fascicularis*) | Normal | **Neuro-EPO** 5750 UI/kg /1000 µl one dose | Pharmacokinetic/security | [86] |
| Mice B6D2F1 | Normal | **Neuro-EPO** 1380–4692 UI/120 µl one dose | Security (potency assays) | [101] |
| Mice B6D2F1 | Normal | **rHu-EPO** 1200–4800 UI/120 µl one dose | Security (potency assays) | [101] |
| Rat | Focal ischemia (MCAO) | **Neuro-EPO** 249.4 UI/10 µl/8 h/2 days | Efficacy | [99] |
| Mice C57 Bl6 | Focal ischemia (MCAO) | **rHu-EPO** 100 UI/12 µl/8 h/4 days | Pharmacokinetic and biodistribution/security | [92] |

MCAO: middle cerebral artery occlusion; UI: International Unit.

The transit of rHu-EPO to the CNS after intranasal administration and its effect were confirmed by us[14]. The detection of the molecule either in the olfactory bulbs or in the cerebellum suggested its contact with the CSF. This was further confirmed by the significant increase of rHu-EPO in the CSF of *M. fascicularis* 5 min after its administration by the intranasal route[86]. In animals treated with Neuro-EPO, a preservation of the habituation behavior in spontaneous exploratory activity was observed in both models, demonstrating the conservation of the structural integrity of the brain regions related to learning, and short- and long-term memory[14].

In the model of MCAO for 2 h in rats, animals treated with Neuro-EPO by the intranasal route displayed smaller volumes of ischemic tissue and a better clinical condition at 48 h[115]. The results of this study in rodents show therapeutic efficacy in both the acute and chronic phases of ischemia, as well as in reperfusion ischemia models, suggesting neuroprotective effects in brain structure and function. These are indirect evidences of the access of Neuro-EPO administered in the amounts equivalent to the therapeutic dose recommended for ischemia by the intranasal route.

Those results suggest additional advantages for the intranasal route, which could be safer and faster than the intravenous route[99,100].

In correlation with these ideas, Fletcher et al.[92] recently demonstrated that intranasal EPO plus IGF-I penetrate into the brain more efficiently than other drug delivery methods, and could potentially provide a fast and efficient treatment to prevent chronic effects of stroke.

A general survey carried out by a group of investigators concerning the use of the intranasal route to administer drugs for the treatment of diseases affecting the CNS indicated that in the last decade, roughly 11% of the new drugs generated by the industry are administered by this route. Patients prefer intranasal administration due to the efficacy and safety of these formulations. This report emphasizes that this type of drug requires a rigorous dose study to demonstrate safety, since it is a very rapid route to the CNS. The characteristics of the excipients, vehicles, preservatives, and packaging for these types of formulations also require further studies, because they can improve the stability of the formulation and maintain the efficacy of the drug previously demonstrated in preclinical studies[101].

## CONCLUSION

Although few scientific groups have approached the nasal administration of EPOs, the results are concurrent. Neuro-EPO is similar to brain-endogenous EPO, and its nasal administration is safer and faster than other routes, circumventing the BBB and its peripheral removal by the liver.

In conclusion, intranasal EPO is a potential, novel, neurotherapeutic approach. The above-mentioned studies suggest the safety and proof of concept for the initiation of clinical trials in stroke patients.

## DISCLOSURE

Drs. García Rodríguez and Sosa Teste are coinventors on the patent “rh-epo nasal formulations with low sialic acid concentration for the treatment of diseases of the central nervous system”, cu. Patent Formulation International Application No.: PCT/CU2006/000007 OCPI, Havana, Cuba. The authors report no other conflict of interest concerning in this paper.

## ACKNOWLEDGMENTS

We thank Charles M. Hollingshead, University of Guelph, and Alina Gonzalez-Quevedo, Neurology Institute of Havana, Cuba, for critical reading of this manuscript.
